# CTAB Enhances Chemo-Sensitivity Through Activation of AMPK Signaling Cascades in Breast Cancer

**DOI:** 10.3389/fphar.2019.00843

**Published:** 2019-07-26

**Authors:** Yue Pan, Yunqiu Zhang, Qing Chen, Xufeng Tao, Jianzhou Liu, Gary Guishan Xiao

**Affiliations:** ^1^School of Chemical Engineering, Dalian University of Technology, Dalian, China; ^2^Functional Genomics and Proteomics Laboratory, Osteoporosis Research Center, Creighton University Medical Center, Omaha, NE, United States

**Keywords:** CTAB, breast cancer, chemosensitivity, AMPK signaling, HIF-1α

## Abstract

Metabolic reprogramming is thought to be one of the initiators in cancer drug resistance. It has been shown that CTAB is capable of interfering the efficiency of cancer therapy by regulation of cell metabolic reprogramming. In this study, we hypothesized that AMPK as a key metabolic regulator plays a crucial role in regulation of breast cancer drug resistance, which could be alleviated by treatment of CTAB. We observed that CTAB can improve the DOX sensitivity of the breast cancer cells by inhibition of the ATP-dependent drug-efflux pump P-gp complex through activation of the AMPK-HIF-1α-P-gp cascades. The CTAB effect was also confirmed *in vivo* showing low systemic toxicity. Taken together, our results showed that CTAB sensitized drug resistance of breast cancer to DOX chemotherapy by activating AMPK signaling cascades both *in vitro* and *in vivo*, suggested that CTAB may be developed as a promising and novel chemosensitizer and chemotherapeutic candidate for breast cancer treatment.

## Introduction

Breast cancer is one of the lethal malignancy in female and has a relative lower survival rate in women (Dey et al., 2019). Although chemotherapy could reduce the size of primary tumor, drug resistance leads severely to an early relaps during therapy, resulting in significant morbidity (Tang et al., 2016). A growing amount of evidence suggests that inhibition of drug-induced apoptosis, the extrusion of drug by cell membrane pumps, redistribution of intracellular accumulation of drugs, modification of drug target molecules, and up-regulation of lipids play crucial roles in chemotherapeutic resistance (Wu et al., 2014). The exact molecular mechanisms underlying DOX-mediated drug resistance of breast cancer are still poorly defined. Therefore, understanding better the pathogenesis of therapeutic resistance is of great importance to develop novel therapeutic strategies to improve the prognosis of breast cancer patients.

Recently, cellular metabolic reprogramming event may be closely related to cancer drug resistance (Bhattacharya et al., 2016). Due to the Warburg effect, cancer cells can maintain its energy homeostasis by averting to the intensified aerobic glycolysis from the mitochondrial oxidative phosphorylation in normal cell physiology (Liberti and Locasale, 2016). Due to cellular metabolic rewiring and reprogramming, the metabolic characteristics (i.e., metabolic phenotypes) were altered, resulting in the changed microenvironment of cancer cells facilitating cancer cells’ apoptotic escape during chemotherapy and radiotherapy (Icard et al., 2018). One of the most prominent mechanisms underlying multiple drug resistance (MDR), for example, is characterized as an over-expression of ATP binding cassette (ABC) transporters associated with the Warburg effect (Yaojie Fu et al., 2017). A study from P-glycoprotein (P-gp), as one of the most studied ATP-dependent drug-efflux pump, showed that it may be an essential obstacle to reducing intracellular drug accumulation in tumor eradication (Joshi et al., 2017). This suggests that P-gp overexpression may be initiated by metabolic reprogramming in order to facilitate cell adaptation to microenvironment changes, such as hypoxia, acidosis, or nutrient starvation (Washio et al., 2019). The AMP-activated protein kinase (AMPK) is a prominent sensor sensing cellular energy status, which the activation of AMPK promotes catabolic processes and inhibits anabolic processes in response to ATP cellular demands (Weidong et al., 2015). AMPK activation may result from the change of microenvironment through metabolic reprogramming (Wang et al., 2019). It has reported that the activation of AMPK consequently inhibits HIF-1α, and HIF-1α is the up-stream of P-gp and contributes to drug resistance in a variety of human tumors (Pan et al., 2017a; Pan et al., 2017b). Inhibition of drug-induced apoptosis or apoptotic escape also plays an important role in drug resistance (Kartal-Yandim et al., 2016). In addition, activation of AMPK signaling cascades could also inhibit tumorigenesis by suppressing ACC1 activity (Chen et al., 2019). CTAB (cetyl trimethyl ammonium bromide) has two parts; it has a nonpolar hydrophobic tail and a positively charged polar head (Zhang et al., 2013). This structure has the ability to increase the permeability of cell membrane, due to the CTAB that could form the nanoscale and attach onto the cell membrane (Ito et al., 2009). The increment of cell membrane permeability is a pivotal event in regulating H^+^-ATP synthase, which could reduce the ATP levels in cancer cells (Zhang et al., 2013). AMPK is the sensor of intracellular energy and is sensitive to the change of AMP to ATP ratio (Tan et al., 2018).

In the present study, the molecular mechanism of CTAB-regulated drug resistance of breast cancer was investigated. We found that CTAB enhanced DOX chemosensitivity of breast cancer mainly through activation of AMPK signaling cascades.

## Materials and Methods

### Chemicals and Reagents

CTAB (H9151-100G, purity ≥ 99%), compound C, and sulforhodamine B were purchased from Sigma-Aldrich. DMEM high-glucose medium and fetal bovine serum (FBS) were purchased from the Gibco (USA). HIF-1α overexpression plasmid was bought from GenePharma (China). The primary antibodies were from Santa, including GAPDH (Cat. # sc-25778), p-AMPK (Cat. # sc-33524), AMPK (Cat. # sc-25792), P-gp (Cat. # sc-55510), and HIF-1α from Abcam (Cat. #113642).

### Cell Culture and SRB Assay

Human breast cancer MCF-7 and human breast cancer multi-drug resistance MCF-7/MDR cell lines were purchased from Central South University. Cells were treated with DOX at final concentrations of 0, 0.31, 0.63, 1.25, 2.5, 5, 10, 20, and 40 μg/ml combined with 0.8 μg/L CTAB, and/or 0.8 μg/L compound C, and/or HIF-1α overexpression plasmid, respectively, for 48 h. An SRB assay was used to assess cell viability.

### Western Blot Analysis

Cell total protein and cytoplasmic protein were extracted, and the concentration was measured by BCA Kit (Solarbio, China). Protein was separated by SDS-PAGE and transferred to PVDF. The PVDF was incubated in blocking buffer (5%BSA) for 2h. The primary antibody was diluted (1:500) by TBST and incubated with PVDF overnight at 4°C. And the second antibody (1:2,000) was incubated with PVDF for 2 h at room temperature. Bands were visualized by an ECL Western Blotting Detection System (Tanon 4200).

### Subcutaneous Transplanted Tumor Model Evaluation in Nude Mice

All experiments were approved by the Animal Care and Use Committee of Dalian University of Technology, and the procedures were performed in strict accordance with the People’s Republic of China Legislation Regarding the Use and Care of Laboratory Animals. MCF-7/MDR cells (1 ×10^6^) were injected in female nude mice (Vital River Laboratories, Beijing, China) subcutaneously. Control group treated with saline injected by tail vein every 3 days, the DOX group treated with DOX (1 mg·kg^−1^) by tail vein injection every 3 days, the CTAB group treated with CTAB (0.5 mg·kg^−1^) by tail vein injection every 3 days, the CTAB combined with DOX group (CTAB+DOX) treated with CTAB (0.5 mg·kg^−1^), and DOX (1 mg·kg^−1^) by tail vein injection every 3 days. The tumor size and body weight were measured every 3 days. The mice were given the drugs for 26 days.

### Statistical Analysis

All data were expressed as the mean ± SD. Significance among groups was analyzed by one-way analysis of variance (ANOVA), followed by Dunnett’s multiple comparison post-test. A value with *p* < 0.05 was considered significant.

## Results

### Characteristics of DOX Resistance of Breast Cancer Cell

**Figure 1:**
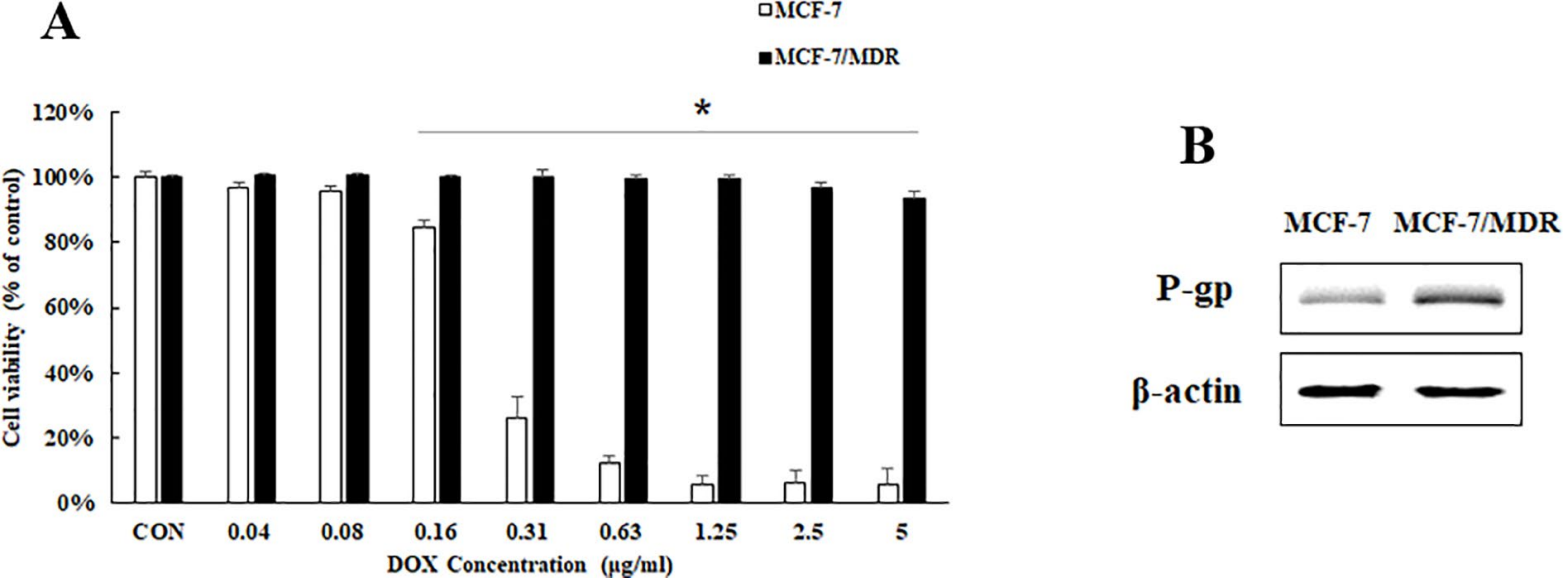
Cell viability and P-gp protein expression in MCF-7 and MCF-7/MDR cells. **(A)** Effect of DOX on cell viability on MCF-7 and MCF-7/MDR cell. Mean values ± SD, *P < 0.05, MCF-7/MDR cells treated with different concentration of DOX for 48 h *vs*. MCF-7 cells treated with different concentration of DOX for 48 h. **(B)** P-gp protein expression in MCF-7 and MCF-7/MDR cells.

In order to investigate the drug resistance of breast cancer, we establish a breast cancer cell line MCF-7/MDR to DOX resistance. To confirm the DOX sensitivity of MCF-7/MDR cells, we treated MCF-7 and MCF-7/MDR with different concentrations of DOX. The MTT results showed that DOX at an initial dose of 0.36 µg/ml inhibited significantly cell proliferation of MCF-7, while DOX did not have any effect on cell proliferation of MCF-7/MDR until it was at an effective dose of 2.5 µg/ml (**Figure 1A**). P-gp (P-glycoprotein) is a known biomarker showing multidrug resistance to therapies. Compared with MCF-7 cell, expression of the P-gp in MCF-7/MDR is higher (**Figure 1B**), showing that MCF-7/MDR cell is a cell line with resistance to DOX, and will be used for this study.

### CTAB Enhances Chemo-Sensitivity of MCF-7/MDR Cell to DOX

**Figure 2:**
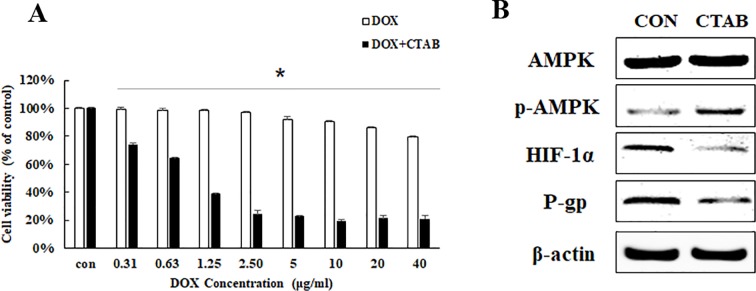
CTAB enhanced DOX sensitivity on MCF-7/MDR cells. **(A)** Effect of DOX and DOX combined with CTAB on cell viability of MCF-7/MDR cells for 48 h. Mean values ± SD, *P < 0.05, MCF-7/MDR cells treated with different concentration of DOX for 48 h vs. MCF-7 cells treated with different concentration of DOX combined with CTAB for 48 h. **(B)** AMPK, p-AMPK, HIF-1α, and P-gp protein expression caused by CTAB treatment.

To confirm the effects of CTAB on the enhancement of DOX sensitivity in breast cancer, MCF-7/MDR cells were treated with DOX at various concentrations from 0.31 to 40 μg/ml for 48 h combined with CTAB at a dose of 0.8 μg/ml. As expected, DOX alone at a dose of 2.5 µg/ml won’t have any inhibitory effects on the cell proliferation while the treatment of DOX combined with CTAB showed significant inhibitory effects of the cell proliferation at a dose of 0.36 µg/ml (**Figure 2A**); compared with the DOX group, the cell viability of combined group is lower (**Figure 2A**). AMPK signaling pathway is involved in the drug resistance. To discuss the mechanism of CTAB enhancing DOX sensitivity, we firstly investigated the protein expression of p-AMPK, HIF-1α, and P-gp; as shown in **Figure 2B**, CTAB induced the AMPK phosphorylation and inhibited HIF-1α and P-gp expression. These data demonstrated that treating MCF-7/MDR cells with CTAB resulted in a significant chemotherapy sensitization effect, and the mechanism may be through regulating the AMPK signaling pathway.

### CTAB Enhances DOX Sensitivity Through Activating AMPK

**Figure 3:**
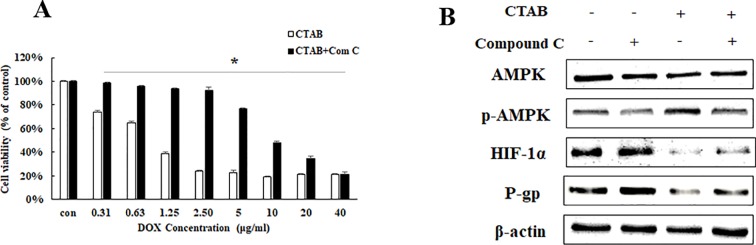
The effect of AMPK on CTAB sensitizing drug resistance. **(A)** Cell viability in MCF-7/MDR cells after DOX combined with CTAB treatment, or DOX combined with CTAB and compound C treatment. Mean values ± SD, *P < 0.05, MCF-7/MDR cells treated with DOX combined with CTAB for 48 h *vs*. MCF-7/MDR cells treated with DOX combined with CTAB and compound C for 48 h. **(B)** AMPK, p-AMPK, HIF-1α, and P-gp protein expression after CTAB treatment or CTAB combined compound C treatment.

To further explore the potential molecular mechanisms of CTAB-related reversing MDR by focusing on the target gene AMPK, we detected the DOX sensitivity and expression of HIF-1α and P-gp expression in MCF-7/MDR cells when AMPK expression was specifically inhibited by compound C (a selective AMPK inhibitor). We examined the effect of an AMPK inhibitor (compound C) on MCF-7/MDR cells. As indicated in **Figure 3A**, in the presence of compound C, the CTAB co-treatment with DOX-elicited anti-proliferative effect was significantly diminished. To address the role of AMPK activation in the inhibitory effect of CTAB on P-gp and HIF-1α expressions, as shown in **Figure 3B**, compound C blocked CTAB-induced AMPK activation and prevented the inhibition of HIF-1α and P-gp caused by CTAB. These findings suggested that CTAB enhanced DOX sensitivity and blocked HIF-P-gp pathway *via* activation of AMPK.

### HIF-1α is the Downstream of AMPK in CTAB-Induced Chemo-Sensitivity Enhancement

**Figure 4:**
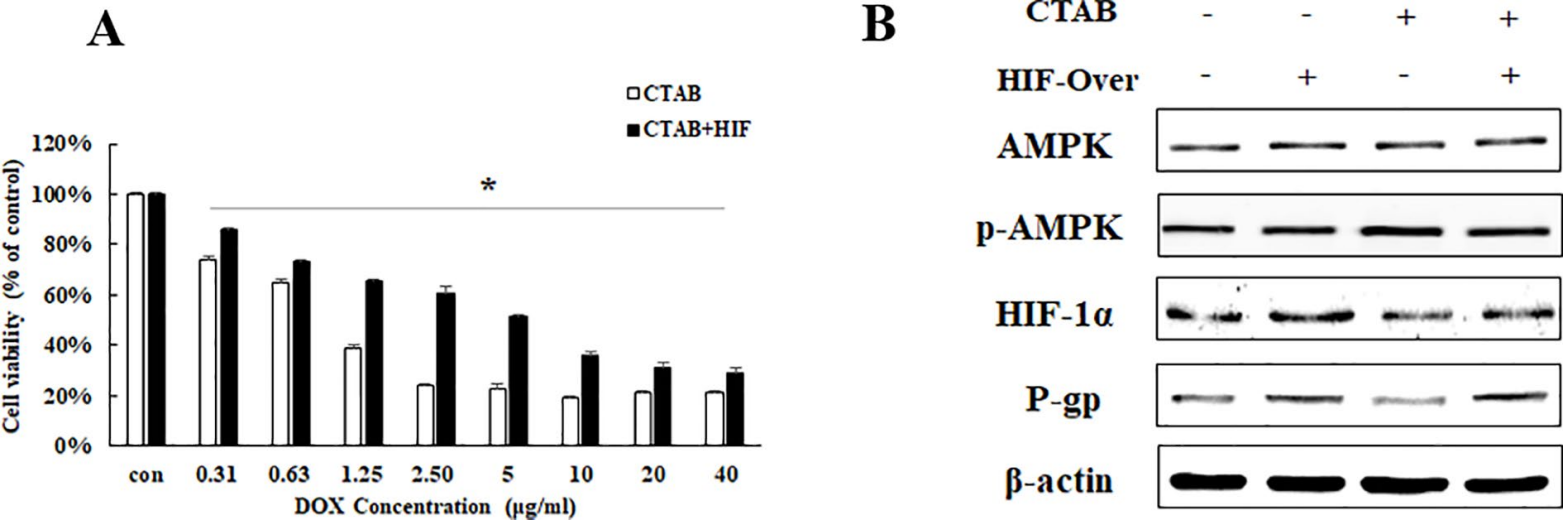
The effect of HIF-1α on CTAB sensitizing drug resistance. **(A)** Cell viability in MCF-7/MDR cells after DOX combined with CTAB treatment, or DOX combined with CTAB and HIF-1α overexpression plasmid treatment. Mean values ± SD, *P < 0.05, MCF-7/MDR cells treated with DOX combined with CTAB for 48 h *vs*. MCF-7/MDR cells treated with DOX combined with CTAB and HIF-1α overexpression plasmid for 48 h. **(B)** AMPK, p-AMPK, HIF-1α, and P-gp protein expression after CTAB treatment or CTAB combined HIF-1α overexpression plasmid treatment.

To further elucidate the relationship between HIF-1α and P-gp in CTAB, HIF-1α overexpression plasmid was added before CTAB exposure. We also found that overexpressing HIF-1α specifically decreased the effect of CTAB-enhanced DOX sensitivity on MCF-7/MDR cell (**Figure 4A**). While treated MCF-7/MDR cells with a combination of HIF-1α overexpression plasmid and CTAB caused a significant increase of HIF and P-gp than CTAB alone (**Figure 4B**). However, overexpressing HIF-1α did not affect the expression of p-AMPK (**Figure 4B**). These ﬁndings suggest that CTAB activates HIF-P-gp pathway to increase DOX sensitivity on MCF-7/MDR cells, and HIF-1α is the downstream of AMPK.

### CTAB Reversed the Multi-Drug Resistance *In Vivo* With Low Side Effects

**Figure 5:**
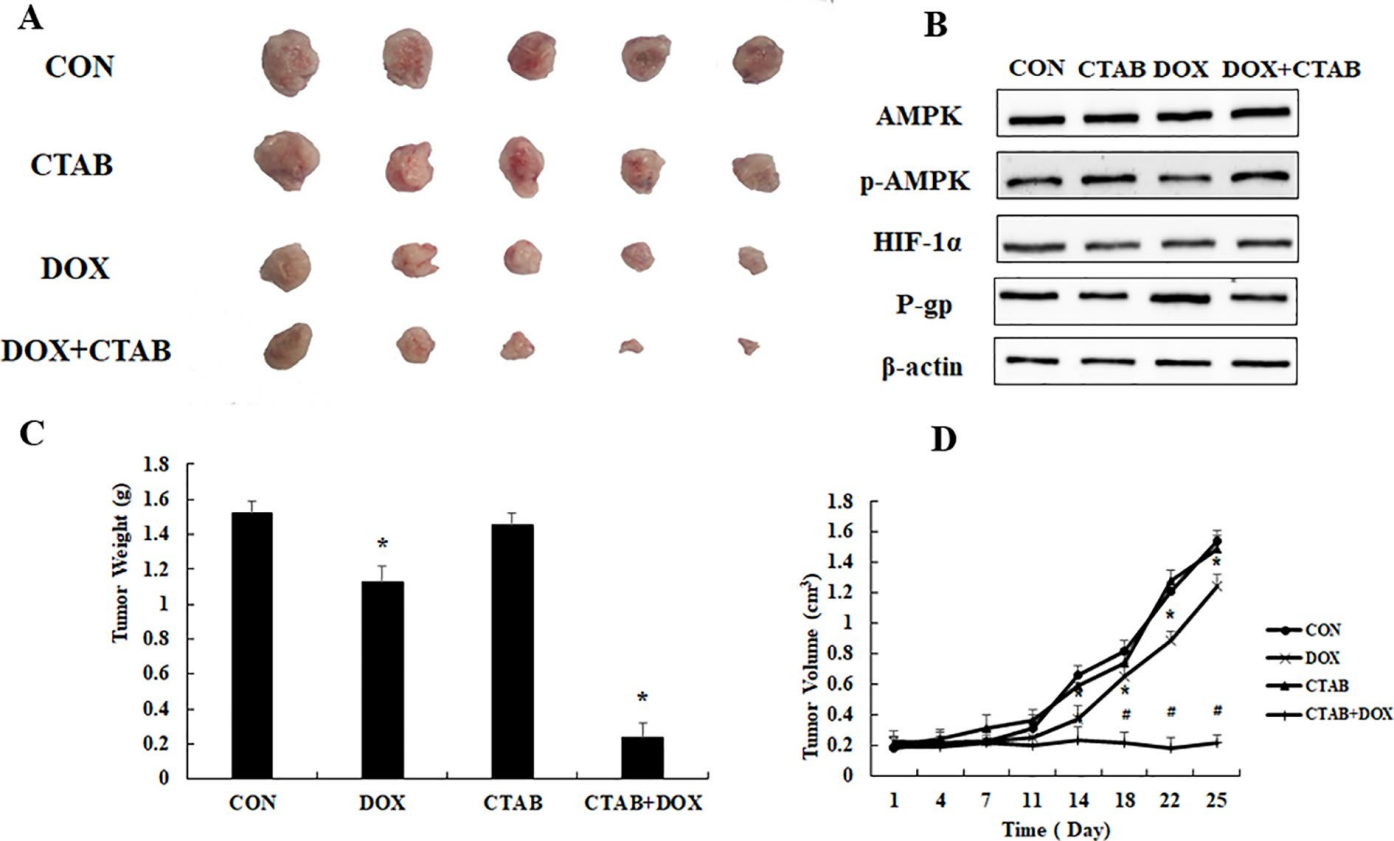
The overcoming of drug resistance effect of CTAB *in vivo*. **(A)** Tumor photographs, **(B)** tumor weight, **(C)** tumor volume. Mean values ± SD, *P < 0.05, *vs*. control group. **(D)** AMPK, p-AMPK, HIF-1α, and P-gp protein expression in MCF-7/MDR xenograft.

To evaluate the overcoming drug resistance effect of CTAB, we generated transplanted drug resistance breast tumor model by subcutaneous inoculation of 1 × 10^6^ MCF-7/MDR cells. **Figures 5A, C** showed that, compared with other groups, the DOX combined CTAB group has the smallest tumor size. Compared to control group, DOX group was effective in delaying tumor growth, but it is not as effective as the combined group mentioned as above. We have investigated the mechanism of CTAB on MCF-7/MDR cells *in vivo*. As shown in **Figure 5D**, CTAB administration significantly induced phosphorylation of AMPK and down-regulated the expression of HIF and P-gp, which are in lined with the *in vitro* data. These data further verified that CTAB-induced AMPK activation reserved drug resistance *in vivo*.

**Figure 6:**
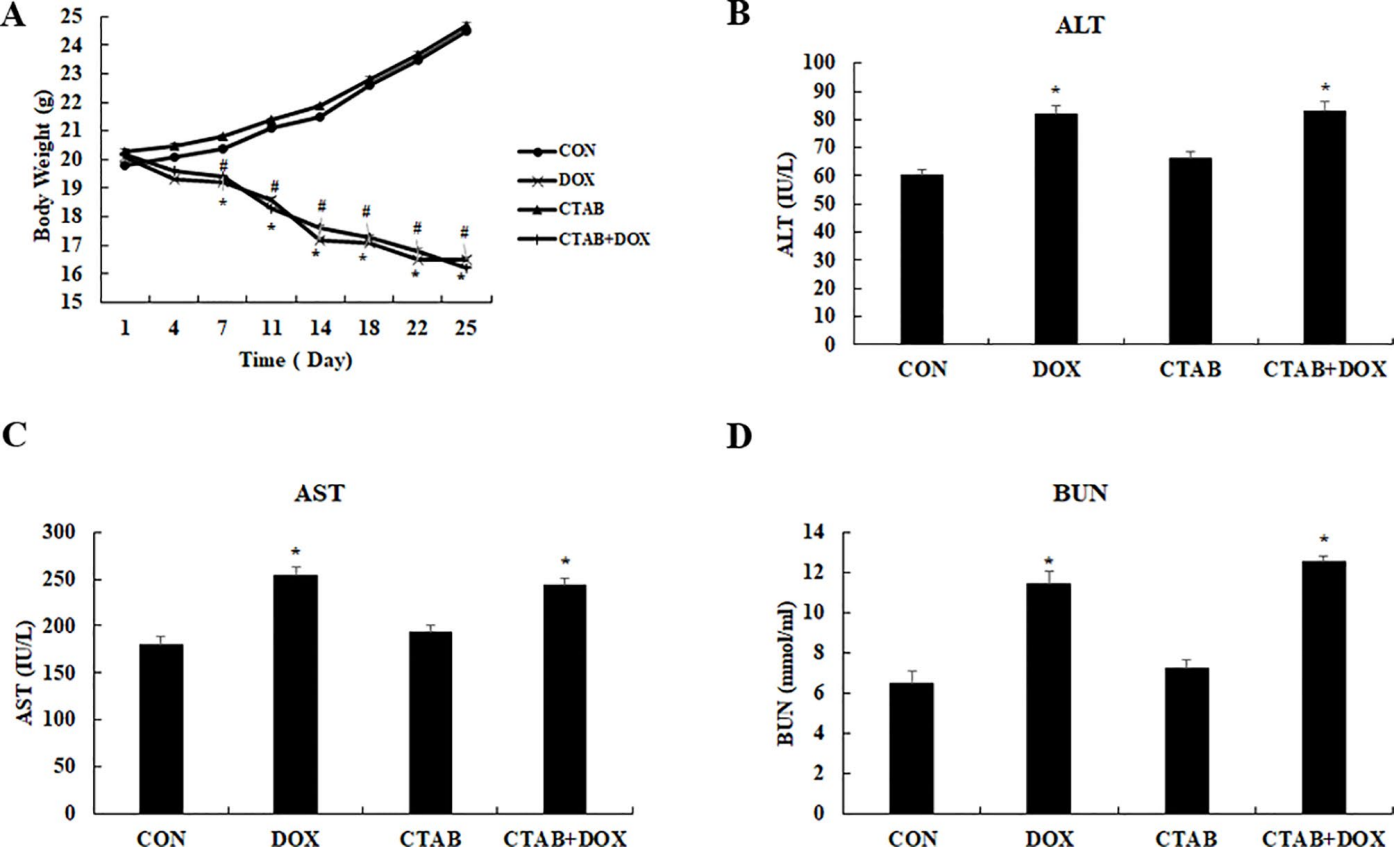
Assessment of the systemic side effects of the different treated mice. **(A)** Body weight **(B)** aspartate aminotransferase (AST), **(C)** blood urea nitrogen (BUN), **(D)** alanine aminotransferase (ALT). Mean values ± SD, *P < 0.05, *vs*. control group.

Finally, we evaluated the systemic safety of the proposed CTAB; the toxicological index including body weight and blood chemistry was analyzed using the breast tumor model. As shown in **Figures 6A, D**, aspartate transaminase (AST), hepatic function enzymes including alanine transaminase (ALT), kidney function enzymes including blood urea nitrogen (BUN) and body weight were observed to evaluate the safety. Compare the control group, all the indexes were significantly increased in DOX and DOX combined with CTAB group. Noticeably, CTAB-only group do not have significant difference with the control group. It is feasible to anticipate that CTAB has the reversing the multi-drug resistance effect and systemic safety.

## Discussion

Our previous and others works showed that CTAB has anti-tumor effect (HCC) and may have positive potential to overcome drug resistance (Wissing et al., 2013; Pan et al., 2015). Moreover, our work showed that CTAB could overcome DOX resistance in breast cancer. Furthermore, in our work, the mice treated with DOX have loss of body weight and abnormal serum indexes. But CTAB showed the safety to overcome drug resistance. CTAB exhibits little resistance and low toxicity to normal tissues during clinically bactericidal therapy. Our observations suggest that CTAB may be potentially useful in therapeutic efforts to overcome breast cancer drug resistance.

Our work reported that CTAB can enhance chemo-sensitivity on cancer cells. The most prominent mechanisms underlying MDR is over-expression of ATP-binding cassette (ABC) transporters. MDR1/P-gp play an important role in drug resistance due to that it could pump the drug out of the cell and reduce the intracellular concentration (Chang et al., 2019). Our results show that CTAB can increase DOX sensitivity to drug resistance of breast cancer *in vitro* and *in vivo* through inhibiting P-gp expression. Recently, some studies showed that P-gp is the down-stream of HIF-1α and played an important role in variety of human tumors (Muz et al., 2017; Wang et al., 2018). Therefore, HIF-1α/MDR1 signaling pathway would be a target for cancer drug resistance therapy (Zhao et al., 2015). Consistent with these reports, our results showed CTAB could inhibit HIF-1α and P-gp expressions. Under HIF-1α overexpression plasmid, the DOX sensitivity caused by CTAB was restored and the P-gp expression has also been reactivated. Recently, study showed the translation of HIF-1α is regulated by p70S6K and 4E-BP1 kinase (Gong et al., 2019). However, 4E-BP1 and p70S6K are the downstream of mTOR signaling (Faton and Jiang, 2013). In addition, AMPK is involved in cancer proliferation, metastasis, and drug resistance through regulating mTOR signaling (Qing Wei et al., 2019).

Besides the sensor of energy, the activating of AMPK takes part in the tumor especially in the drug resistance (Wang et al., 2010; Xiaoguang et al., 2018). A serious of compound has pharmacological effect on tumor through activating AMPK. Emerging evidence has demonstrated the potent anti-tumor effect of biguanides, among which phenformin was reported to potentially be a more active anti-cancer agent than metformin (Liu et al., 2015). The first evidence of the anti-proliferative effect of phenformin was obtained in cancer cells in 2003 (Caraci et al., 2003). It was then demonstrated that phenformin significantly inhibited both the development and growth of MCF-7 tumors through the activation of AMPK pathway (Appleyard et al., 2012; Liu et al., 2015).

AMPK is the sensor of energy metabolism. CTAB could penetrate mitochondrial membranes and accumulate in the mitochondrial in response to the negative transmembrane potential. Thus, CTAB would be predicted to be more effective against tumors that rely heavily on glycolysis and are dependent on the Warburg effect (Emma et al., 2009). Our results showed that CTAB could activate AMPK. Furthermore, compound C, the inhibitor of AMPK, led to an enhanced expression of HIF-1α and P-gp. Furthermore, it reduced the DOX sensitivity caused by CTAB, suggesting a negative correlation between the two molecules. Our study has demonstrated that the enhancement chemo-sensitivity effects caused by CTAB were mediated by down-regulating HIF-1α-P-gp pathway expression through activating AMPK.

In summary, we observed that CTAB-induced AMPK activation reserved drug resistance *in vitro* and *in vivo* with low systemic toxicity. Our study has demonstrated that CTAB enhances DOX sensitivity through AMPK-HIF-1α-P-gp pathway. Indeed, our study shed light on a new AMPK targeting strategy in drug resistance breast cancer treatment based on a CTAB to achieve the most efficient and safe therapy.

## Conclusion

In conclusion, CTAB, as a compound for nanoparticle synthesized, has the potential of overcoming drug resistance in breast cancer. CTAB could enhance the DOX sensitivity in breast cancer *in vivo* and *in vitro*. The mechanism may be activating the AMPK and then down-regulating HIF-1α-P-gp pathway. Besides, the CTAB showed high systemic safety and could serve as a multidrug resistance reverser for future breast cancer therapy.

## Ethics Statement

This study was carried out in accordance with the recommendations of Dalain University of Technology. The protocol was approved by the Dalain University of Technology.

## Author Contributions

All authors conceived and designed the study. YP, YZ, and QC performed the experiments. YP, XT, JL, and GX analyzed and interpreted the data, and prepared the manuscript.

## Funding

This work is sponsored by National Natural Science Foundation of China (81803024, 81770846) and Fundamental Research Funds for the Central Universities under Grant No. DUT17RC(3)068.

## Conflict of Interest Statement

The authors declare that the research was conducted in the absence of any commercial or financial relationships that could be construed as a potential conflict of interest.
